# HIF2 Regulates Intestinal Wnt5a Expression

**DOI:** 10.3389/fonc.2021.769385

**Published:** 2021-11-25

**Authors:** Carolina J. García García, Ariana C. Acevedo Diaz, Neeraj Kumari, Suman Govindaraju, Marimar de la Cruz Bonilla, F. Anthony San Lucas, Nicholas D. Nguyen, Iancarlos Jiménez Sacarello, Helen Piwnica-Worms, Anirban Maitra, Cullen M. Taniguchi

**Affiliations:** ^1^ The University of Texas MD Anderson Cancer Center UTHealth Graduate School of Biomedical Sciences, Houston, TX, United States; ^2^ Department of Radiation Oncology, The University of Texas MD Anderson Cancer Center, Houston, TX, United States; ^3^ Department of Experimental Radiation Oncology, The University of Texas MD Anderson Cancer Center, Houston, TX, United States; ^4^ School of Medicine, University of Puerto Rico, Rio Piedras, PR, United States; ^5^ Department of Biology, University of Puerto Rico, Bayamon, PR, United States; ^6^ Department of Pathology, The University of Texas MD Anderson Cancer Center, Houston, TX, United States

**Keywords:** radiotherapy, intestinal stem cells, GI radiotoxicity, hypoxia, HIF2, Wnt5a

## Abstract

Radiation therapy for abdominal tumors is challenging because the small intestine is exquisitely radiosensitive. Unfortunately, there are no FDA-approved therapies to prevent or mitigate GI radiotoxicity. The EGLN protein family are oxygen sensors that regulate cell survival and metabolism through the degradation of hypoxia-inducible factors (HIFs). Our group has previously shown that stabilization of HIF2 through genetic deletion or pharmacologic inhibition of the EGLNs mitigates and protects against GI radiotoxicity in mice by improving intestinal crypt stem cell survival. Here we aimed to elucidate the molecular mechanisms by which HIF2 confers GI radioprotection. We developed duodenal organoids from mice, transiently overexpressed non-degradable HIF2, and performed bulk RNA sequencing. Interestingly, HIF2 upregulated known radiation modulators and genes involved in GI homeostasis, including *Wnt5a*. Non-canonical Wnt5a signaling has been shown by other groups to improve intestinal crypt regeneration in response to injury. Here we show that HIF2 drives *Wnt5a* expression in multiple duodenal organoid models. Luciferase reporter assays performed in human cells showed that HIF2 directly activates the *WNT5A* promoter *via* a hypoxia response element. We then evaluated crypt regeneration using spheroid formation assays. Duodenal organoids that were pre-treated with recombinant Wnt5a had a higher cryptogenic capacity after irradiation, compared to vehicle-treated organoids. Conversely, we found that *Wnt5a* knockout decreased the cryptogenic potential of intestinal stem cells following irradiation. Treatment with recombinant Wnt5a prior to irradiation rescued the cryptogenic capacity of *Wnt5a* knockout organoids, indicating that Wnt5a is necessary and sufficient for duodenal radioprotection. Taken together, our results suggest that HIF2 radioprotects the GI tract by inducing Wnt5a expression.

## Introduction

Radiation is one of the four pillars of cancer care, with approximately half of cancer patients receiving radiotherapy at some point of their treatment regimen (1). Similar to chemotherapy, the efficacy of radiotherapy is limited by normal tissue toxicity. This toxicity is especially limiting in the case of abdominal and pelvic cancers, which are surrounded by the exquisitely radiosensitive gastrointestinal (GI) tract, and they require high doses of radiation to achieve tumoricidal effects (2). Multiple studies have highlighted how common GI radiotoxicity is among cancer patients (3–5). Results from a Phase 3 clinical trial showed that over a third of patients treated with 45 Gy or 50.4 Gy four-field pelvic radiotherapy or pelvic intensity-modulated radiotherapy reported GI symptoms following radiation treatment (6). Consequently, abdominal radiotherapy for cancers of the hepatobiliary tract and pancreas are administered at sub-curative doses to avoid GI radiotoxicity. Recent clinical trials have shown that dose-escalated radiotherapy using highly precise 3D conformal radiation techniques, such as stereotactic body radiotherapy and intensity-modulated radiotherapy, can improve outcomes (7–9), but these techniques still cannot fully avoid the small intestines (10). Furthermore, these sophisticated techniques are not widely accessible, as they require specialized expertise that is limited to some academic centers. Thus, an alternative to reduce GI radiotoxicity in cancer patients is to use a radioprotector to prevent radiation-induced damage and/or to improve GI repair following radiotherapy (9).

There are currently no FDA-approved radioprotectors of the GI tract. The intestine has a physiological hypoxia gradient that arises due to its vascular anatomy, and the hypoxia-inducible factors (HIFs) regulate various genes required for intestinal barrier function (11). The HIFs are transcription factors which are hydroxylated by the EGLN family of prolyl hydroxylases in the presence of oxygen, iron, and 2-oxoglutarate, allowing the von Hippel-Lindau E3 ubiquitin ligase complex to bind and tag HIFs for proteasomal degradation (12–19). The stabilization of HIFs through hypoxia or EGLN inhibition allows them to regulate cell metabolism and survival (12), induce tissue remodeling (20), increase epithelial integrity (21), and promote stem cell survival (13). There are two main HIF isoforms: HIF1 and HIF2. Our group has previously shown that stabilization of HIF2, but not HIF1, significantly reduces GI radiotoxicity without sparing hypoxic pancreatic tumors (22, 23). However, the mechanisms by which HIF2 confers radioprotection to the small intestine remain unclear.

In the current study, we generated a 3D murine small intestinal organoid model system (24), transiently overexpressed a non-degradable *HIF2* allele (25), and performed whole transcriptomic analysis to gain insight in this regard. We found that HIF2 directly induces Wnt5a expression, a non-canonical Wnt family glycoprotein, in both murine and human cell lines, by activating its promoter. Like other Wnt family members, Wnt5a plays an important role in the in the embryonic development and subsequent homeostasis GI tract, and enhances regeneration following injury (26, 27). Here, we show that Wnt5a is necessary for small intestinal crypt regeneration following radiation and that addition of exogenous Wnt5a to duodenal 3D organoid cultures improves their cryptogenic capacity. Together, our data indicate that HIF2 radioprotects the small intestine, at least in part, by inducing Wnt5a expression.

## Materials and Methods

### Cell Lines and Reagents

L-WRN cells were obtained from the ATCC (CRL-3276). HEK293-derived Adherent-293 (AD-293) cells were obtained from Stratagene (240085). CBRLuc-mCherry reporter murine duodenal organoids were a gift from Dr. Helen Piwnica-Worms (28). Murine duodenal organoids were cultured at 37°C in 5% CO_2_ and 5% O_2_, while all other cell lines were cultured at 37°C in standard 5% CO_2_ incubators. All cell lines were authenticated by short tandem repeat profiling and were confirmed to be *Mycoplasma* free. Recombinant human/mouse Wnt5a was purchased from R&D Biosystems (645-WN-010-CF).

### Mice

All experimental mouse work adhered to the standards articulated in the Animal Research: Reporting of *In Vivo* Experiments guidelines. Additionally, all mouse work was approved by the Institutional Animal Care and Use Committee of The University of Texas MD Anderson Cancer Center. Mice were maintained on a 12-hour light/dark cycle and were provided with sterilized water and standard rodent chow (Prolab Isopro RMH 3000 irradiated feed) *ad libitum*. C57BL/6 mice (RRID : IMSR_JAX:000664), *Wnt5a^fl/fl^* mice (RRID : IMSR_JAX:026626) (29), and *R26-LSL-hHIF2a^dPA^* mice (RRID : IMSR_JAX:009674) (25) were obtained from Jackson Laboratories.

### Generation of 3D Small Intestinal Organoids

L-WRN conditioned media was prepared as previously described (24, 28). Briefly, L-WRN cells were maintained in DMEM high glucose media (Sigma, D6429) supplemented with 10% (v/v) FBS (Sigma, F4135, 1% (v/v) Penicillin/Streptomycin (Sigma, P4333), 500 μg/ml Hygromycin B Gold (*In vivo*Gen, ant-hg-1), and 500 μg/ml G418 (Sigma, G8168). Once cells were confluent, the media was replaced with Advanced DMEM/F12 media (Gibco, 12634010) supplemented with 10% (v/v) FBS, 1% (v/v) Penicillin/Streptomycin, and 2 mM L-glutamine (Sigma, G7513). Conditioned media was collected for six days, centrifuged at 3,000 rpm for 5 minutes, vacuum filtered through a 20 μM PES membrane (Thermo Scientific, 567-0020), and stored at -80°C.

Duodenal crypts were isolated from C57BL/6 mice, *R26-LSL-hHIF2a^dPA^* mice, and *Wnt5a^fl/fl^* mice, and 3D organoid cultures were established as previously described (24, 28). For all steps in this protocol, EDTA (Sigma-Aldrich, E7889) was added fresh to both PBS (Cytiva, SH30256.LS) and HBSS without calcium and magnesium (Gibco, 14025092) to a final concentration of 2 mM, and kept on ice. Mice were humanely euthanized by CO_2_ inhalation followed by cervical dislocation. The duodenum was measured 1 cm below the pylorus, and 4 cm were resected and flushed with PBS/EDTA, then incubated on fresh PBS/EDTA for 10 min on ice, and finally transferred to ice-cold HBSS/EDTA. Duodenal samples were then serially vortexed at 1,600 rpm at 4°C, in fresh HBSS each time, for 5 min, 3 min, and 8 min. Supernatants from the second and third vortexes were combined and passed through 70-μm strainers (Corning, 431751) to isolate crypts and remove any villi that might remain after the washes. Duodenal crypts were pelleted at 1,000 rpm at 4°C, then washed in Advanced DMEM/F12 supplemented with 10% (v/v) FBS, 1% (v/v) Penicillin/Streptomycin, and 2 mM L-glutamine, and re-centrifuged as before. The pelleted crypts were resuspended in 50% (v/v) Matrigel (Corning, 354234) diluted with the crypt washing media, and then were seeded as domes into 24-well plates (Corning, 3524). After Matrigel solidification at 37°C, the duodenal organoids were cultured in 50% (v/v) L-WRN conditioned media supplemented with 10 mM Y27632 (ROCK inhibitor; Sigma-Aldrich, Y0503) and 10 μM SB431542 (TGF-β RI Kinase Inhibitor VI; Sigma-Aldrich, 616461). The culture media was refreshed every other day and the organoids were passaged every third day.

### Adenoviral Transduction

Ad-GFP (VVC-U of Iowa-4, Ad5CMVeGFP), Ad-Cre (VVC-U of Iowa-5, Ad5CMVCre), and Ad-Cre-GFP (VVC-U of Iowa-1174, Ad5CMVCre-eGFP) viral vectors were provided by the University of Iowa Viral Vector Core (http://www.medicine.uiowa.edu/vectorcore). Ad-human HIF1 and Ad-human HIF2 viral vectors were previously produced (30) using hHIF1 (Addgene #18955) and hHIF2 plasmids (Addgene #18956) that contain double proline-to-alanine substitutions which render them nondegradable by VHL (31). Duodenal organoids were transduced after at least three passages. First, organoids were harvested by incubation with Cell Recovery Solution (Corning, 354253) for 30 min on ice, then centrifuged at 1,000 rpm for 5 min at 4°C, and then washed with cold PBS. Organoids were then digested into single intestinal stem cells (ISCs) *via* mechanical digestion while incubating in TrypLE (Gibco, 12605010) supplemented with 10 μM Y27632 and 500 μM N-acetylcysteine (Sigma-Aldrich, A0737) for 5 min at 37°C. To neutralize TrypLE, cold Advanced DMEM/F12 media supplemented with 10% (v/v) FBS, 1% (v/v)  Penicillin/Streptomycin, 2 mM L-glutamine, 10 μM Y27632, and 10 μM SB431542 was added and the ISC suspension was centrifuged at 1,500 rpm for 5 min at 4°C. Single ISCs were resuspended in Advanced DMEM/F12 supplemented as described above and passed through a 35-μM strainer (Corning, 352235). Single ISCs were stained with Trypan Blue (Bio-Rad, 1450021) and counted on a TC20 Automated Cell Counter (Bio-Rad). Duodenal ISCs were transduced with adenoviral particles at MOI rates of 50 particles per cell for 1.5 h at 37°C. Afterwards, transduced ISCs and leftover adenoviral particles were resuspended in 50% (v/v) Matrigel, seeded into 24-well plates, and cultured in 50% (v/v) L-WRN conditioned media supplemented with 10 μM Y27632, 10 μM SB431542, and 10 mM Nicotinamide (Sigma-Aldrich, N3376). RNA purification of duodenal organoids was done approximately 72 hours post-transduction.

### Western Blotting

Duodenal organoids were released from Matrigel by incubation with Cell Recovery Solution for 30 min on ice, then centrifugation at 1,000 rpm for 5 min at 4°C, followed by washing with cold PBS. Organoids were then lysed in M-PER mammalian protein extraction reagent (Thermo Fisher Scientific, 78501) supplemented with protease inhibitor (Roche Life Science, 11836170001) and phosphatase inhibitor (Roche Life Science, 4906837001), and then denatured with 4x Laemmli sample buffer (Bio-Rad, 1610747). Equal cell protein lysate amounts were resolved on SDS-PAGE gel (Bio-Rad, 4568034) and transferred onto a nitrocellulose membrane (Bio-Rad, 1620215) using a Trans-Blot Turbo transfer system (Bio-Rad). After blocking with 5% (w/v) skim milk powder (Bio-Rad, 1706404XTU) in TBS-T for 1h at room temperature, the membrane was probed with primary antibodies diluted in Superblock T20 (ThermoFisher Scientific, 37536) at 4°C overnight. Anti-HIF2α rabbit (Novus Biologicals, NB100-122) was used at 1:500 and anti-β-actin rabbit (Cell Signaling Technology, 4970S) was used at 1:1,000. The membrane was then washed and incubated with HRP-conjugated secondary antibody for 1h. The membrane was developed with Clarity Western ECL Substrate kit (Bio-Rad, 1705061) and visualized using a ChemiDoc imaging system (Bio-Rad). Relative protein expression was quantified based on band intensity using ImageJ software (RRID : SCR_003070) and normalized to control group.

### RNA Purification and Quantitative Real Time-PCR Analysis

Duodenal organoids were released from Matrigel as indicated above, then homogenized by vortexing and vigorously pipetting, and then RNA was purified using an RNeasy mini kit following the manufacturer’s handbook (Qiagen, 74106). Reverse transcription was performed with a mix of random primers and oligos using an iScript cDNA Synthesis kit (Bio-Rad, 1708891). Next, qRT-PCR was performed using SYBR Green Master mix (Bio-Rad, 1725124) and the primer assays listed on **Supplementary Table 1**, on a CFX384 Real-time system (Bio-Rad). Relative gene expression and fold change was calculated using *Hprt* and *Tbp* as reference genes.

### RNA Sequencing and Analysis

Duodenal organoids from C57BL/6 mice transduced with either Ad-GFP, Ad-hHIF1, or Ad-hHIF2 were harvested and RNA was purified using an RNeasy mini kit. RNA purity and concentration were measured using an Epoch Microplate Spectrophotometer with a Take3 Micro-Volume Plate, and Gen5 software (v2), all from BioTek Instruments, Inc. Library preparation and sequencing were performed in the Sequencing and Microarray Facility at MD Anderson Cancer Center. The raw sequencing data was downloaded from the core server and low-quality reads were removed and Q20 and GC contents were calculated. Transcript abundance was quantified using the RSEM software package (RRID : SCR_013027) (32). Differential expression analysis was performed using EBSeq software package (RRID : SCR_003526) (33). **Supplementary Tables 2** and **3** show the output differential expression gene matrix for Ad-HIF2 and Ad-HIF1, respectively, compared to Ad-GFP.

### *WNT5A* Promoter Analysis and Dual Luciferase Assays

The human WNT5a promoter from −2000 to +200 nucleotides from the +1 transcriptional start site (34) was synthesized directly into the KpnI- and XhoI-flanked region of the pUC57 vector (Genscript, SD1176). All mutant promoters were synthesized in a similar fashion. The specific sequences of the mutations can be found in **Supplementary Figure 2**. The KpnI- and XhoI-flanked fragments of each of the promoter constructs were excised and then ligated into the compatible BglII- and HindIII-flanked sites in the pGL4.10[*luc2*] vector (Promega, E6651). AD-293 cells were cultured in DMEM supplemented with 10% FBS and 2 mM L-Glutamine. Cells were transfected using Xfect Transfection Reagent (TakaraBio, 631318) using the manufacturer’s standard protocol. For instance, a 96-well opaque plate was seeded with 9.5 x 10^3^ cells/well and transfected with 0.08 μg of hHIF1 plasmid, hHIF2 plasmid, or control GFP plasmid (Addgene #26822), 0.09 μg of *WNT5A*-luciferase construct, and 9 ng of *Renilla* luciferase, along with Xfect polymer. The transfected cells were incubated for 48 h in Opti-MEM media (Gibco, 31985070). Dual luciferase assays were performed using the Dual-Glo^®^ Reagent (Promega, E2940) kit according to manufacturer’s instructions. The luminescence data was measured on a Cytation 3 luminometer (Biotek Instruments, Inc.). Luciferase signal was normalized to Renilla signal, and then *WNT5A* promotor transactivation was calculated as a fold change over control GFP plasmid transfection.

### Spheroid Formation Assays

Spheroid Formation Assays were performed as previously described (28), using a murine duodenal Click Beetle Red Luciferase-mCherry (CBRLuc-mCherry) reporter organoid line and *Wnt5a^fl/fl^* duodenal organoids. Briefly, duodenal organoid cultures were exposed to the indicated pre-radiation treatments and then irradiated using an X-Rad 320 cell irradiator (Precision X-Ray). Immediately after irradiation, the duodenal organoids were harvested using Cell Recovery Solution and then digested into single ISCs using TrypLE supplemented with Y27632 and N-Acetylcysteine, followed by filtering through a cell strainer as detailed above for Adenoviral Transduction. Live cells were quantified using ViaStain™ AO/PI Staining Solution (Nexcelom, CS2-0106) in a Cellometer^®^ Vision CBA Image Cytometer (Nexcelom). Live duodenal ISCs were seeded in Matrigel in 24-well culture plates at a density of 5,000 cells/well. Organoids were maintained in 50% L-WRN conditioned media supplemented with 10 μM Y27632 and 10 μM SB431542 for six days, then surviving cells were assessed *via* bioluminescence or z-stack imaging. For quantification of bioluminescence, organoids were incubated with 300 μg/ml of D-Luciferin Firefly (Gold Biotechnology, L-123) in the media for 20 min at 37°C, and measurements were taken using a CLARIOstar plate reader (BMG Labtech). Sample wells were first normalized to blank Matrigel wells, and then to unirradiated controls. Z-stack images covering the entire Matrigel dome area were taken using a Cytation 3 Cell Imaging Multi-Mode Reader (Biotek Instruments, Inc.). Z-stack images were stitched using Photoshop version 19.1.7 (RRID : SCR_014199) and viable organoids measuring at least 150 μm were manually quantified using ImageJ version 1.52q (RRID : SCR_003070). For organoids analyzed *via* z-stack imaging, the surviving fraction was calculated as described previously (35).

### Statistical Methods

All statistical analyses were performed using GraphPad Prism^®^ V.8 (San Diego, CA; RRID : SCR_002798), with a significance level of α = 0.05. Dunnett’s multiple comparisons test was used to analyze Dual Luciferase Assays.

## Results

### Transcriptomic Analysis of HIF2 Overexpression in Small Intestinal Organoids by RNA Sequencing

**Figure 1:**
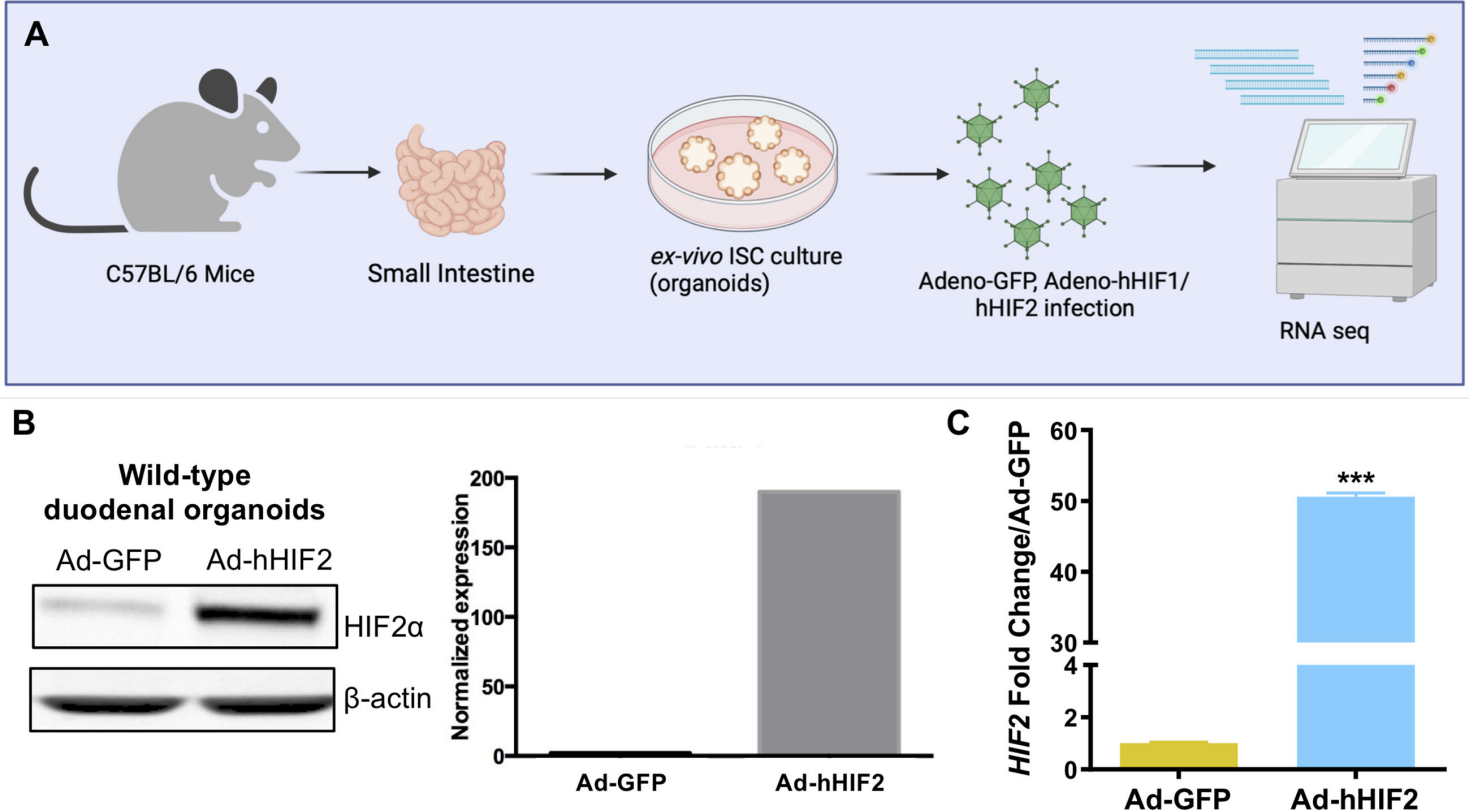
HIF2 overexpression in ISC-enriched duodenal organoid cultures. **(A)** Experimental design to generate 3D murine small intestinal organoid model system. Duodenal organoids were transduced with Adenovirus-human HIF1 (Ad-hHIF1), Ad-hHIF2, or Ad-GFP as control, and then RNA sequencing was performed. **(B)** Validation of adenovirus-mediated gene transfer by Western blot (*left*) and its quantification (*right*). **(C)** Validation of adenovirus-mediated gene transfer by qRT-PCR. All error bars represent mean ± SEM. ***P < 0.001, by Student’s *t* test.

To gain insight into how the HIFs regulate GI biology and to understand the mechanisms by which HIF2 confers radioprotection to the small intestine, we generated a 3D murine small intestinal organoid model system. We harvested duodenum crypts of wild-type C57BL/6 mice, seeded them in basement membrane matrix (Matrigel), and cultured them in conditioned media containing Wnt3a, R-spondin 3, and noggin (**Figure 1A**) (24). These conditions enrich for intestinal stem cells (ISCs), which self-assemble into crypt-like 3D structures that are able to recapitulate the small intestinal crypt *ex vivo* (36). We then transiently overexpressed a non-degradable *HIF1* or *HIF2* allele (25), which contain mutations in the two key prolyl residues that are hydroxylated by the EGLN proteins, rendering them stable even under normoxic conditions, using adenoviral particles (**Figure 1A**). We confirmed adenoviral-mediated HIF overexpression by both western blot and qRT-PCR, and then performed RNA sequencing (**Figures 1A–C**).

**Figure 2:**
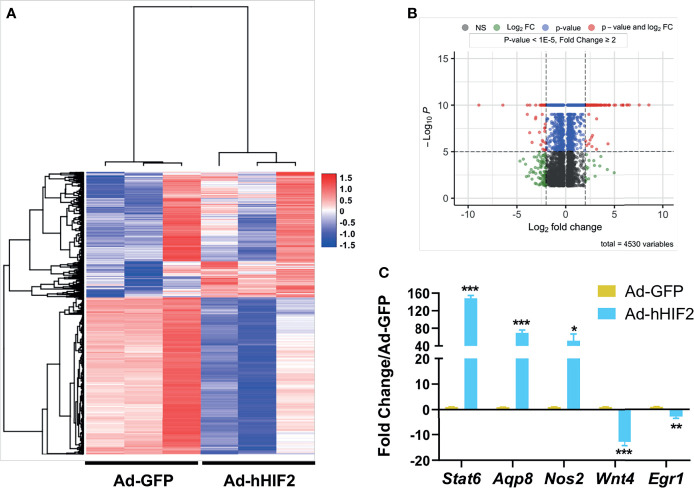
Differentially expressed gene profile in HIF2-overexpressing duodenal organoids. **(A)** Heatmap shows all the differentially expressed genes in duodenal organoids infected with Ad-hHIF2 or control Ad-GFP evaluated *via* RNA sequencing (n = 3 biological replicates/group). **(B)** Volcano plot of the differentially expressed genes. **(C)** qRT-PCR validation of the sequencing data in **(A)**. All error bars represent mean ± SEM. *P < 0.05, **P < 0.01, ***P < 0.001, by Student’s *t* test.

Among thousands of coding transcripts assessed, 1,113 genes exhibited significant differential expression between control GFP- and HIF2-overexpressing duodenal organoids (**Figure 2A** and **Supplementary Table 2**). All the differentially expressed genes with statistical significance were selected with *p* < 0.05, False Discovery Rate (FDR) < 0.05, and at least 2-fold change. Hierarchical clustering analysis revealed a total of 461 upregulated genes and 652 downregulated genes (**Supplementary Table 2**). The entire set of differentially expressed genes can be visualized in a volcano plot in **Figure 2B**. To validate our RNA sequencing results, we independently assessed the expression of 5 genes by qRT-PCR. Our results confirmed that *Stat6*, *Aqp8*, and *Nos2* were upregulated and that *Wnt4* and *Egr1* were downregulated in HIF2-overexpressing duodenal organoids compared to wild-type duodenal organoids, indicating that the RNA sequencing dataset is reliable (**Figure 2C**).

We also examined the transcriptome of HIF1-overexpressing organoids to gain more insight into how the HIFs regulate overall ISC biology. Among the thousands of coding transcripts assessed, only 55 genes exhibited significant differential expression between control GFP- and HIF1-overexpressing duodenal organoids, in which 25 genes were upregulated and 30 genes were downregulated (**Supplementary Figure 1** and **Supplementary Table 3**). Interestingly, the only genes commonly upregulated by HIF1 and HIF2 in our dataset were *Nanp*, *Ppp1r3c*, *Rasgrf1*, *Klhl3*, *Mical2*, and *Nos2*, and the only transcripts commonly downregulated by both HIFs were *Slc18a1*, *Gm37069*, *Hr*, *Gm12480*, *Gm13151*, *Gm21981*, *Klhl30*, *Vcp-rs*, *Gm4204*, *AY512931*, and *Gm28037* (**Supplementary Tables 2** and **3**). We then focused our attention on the HIF2 dataset, as HIF2 and not HIF1, has been shown to be the main HIF isoform driving GI radioprotection (22).

### HIF2 Induces Intestinal Non-Canonical *Wnt5a* Expression

**Figure 3:**
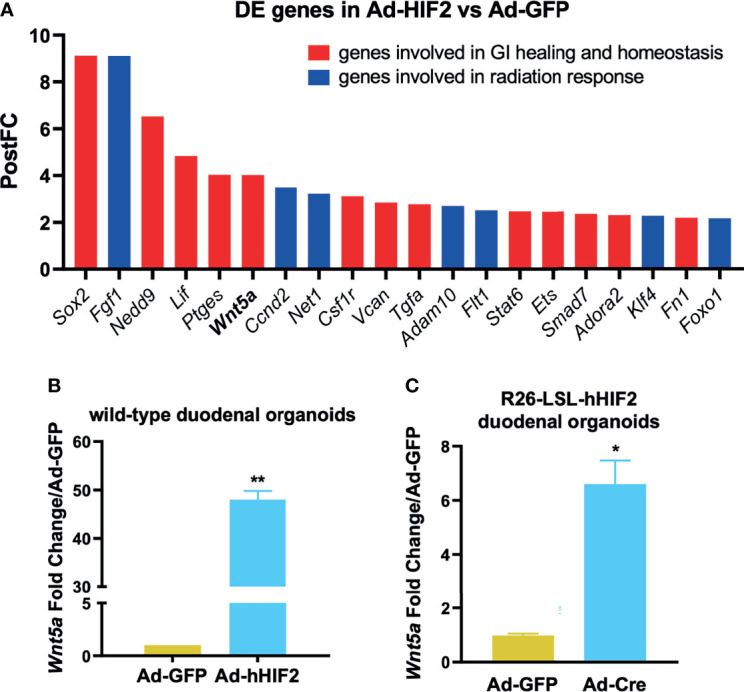
Candidate approach identifies Wnt5a as direct HIF2 target. **(A)** Post-Fold Change (PostFC) of 20 differentially expressed (DE) genes identified from the RNA sequencing analysis. **(B)** qRT-PCR validation of HIF2-induced *Wnt5a* upregulation in wild-type duodenal organoids infected with Ad-GFP or Ad-hHIF2. Data represents 3 biological replicates (3 technical replicates/mouse). **(C)** qRT-PCR showing HIF2-induced upregulation of *Wnt5a* in *LSL-hHIF2* duodenal organoids infected with Ad-GFP or Ad-Cre. Data represents 3 biological replicates (3 technical replicates/mouse). All error bars represent mean ± SEM. *P < 0.05, **P < 0.01, by Student’s *t* test.

Interestingly, the HIF2-induced transcriptome included known radiation modulators as well as genes involved in GI healing and homeostasis, as highlighted in **Figure 3A** in blue and red, respectively. We identified *Wnt5a* as a transcriptional target of HIF2, but not HIF1 (**Figure 3A** and **Supplementary Tables 2** and **3**), and interestingly, Wnt5a has a known connection to non-canonical intestinal crypt regeneration (26). Thus, we took a candidate approach to further investigate its transcriptional regulation by HIF2. We verified that *Wnt5a* was upregulated by HIF2 using two approaches. First, we performed qRT-PCR to independently evaluate duodenal organoids that transiently overexpressed non-degradable *HIF2 via* Adeno-hHIF2 transduction (25), and found significantly increased *Wnt5a* expression by almost 50-fold compared to organoids transduced with Adeno-GFP (**Figure 3B**). Second, we generated duodenal organoids from mice that conditionally overexpressed non-degradable human *HIF2* by knock-in into the *Rosa* 26 *locus* (R26-LSL-hHIF2) (25), transduced them with either Adeno-Cre or control Adeno-GFP vectors, and assessed *Wnt5a* expression using qRT-PCR. Stable *HIF2* overexpression also resulted in significant upregulation of *Wnt5a* by 6-fold (**Figure 3C**). Together, these results suggest that HIF2 induces intestinal *Wnt5a* expression.

### HIF2 Directly Activates the WNT5A Promoter

**Figure 4:**
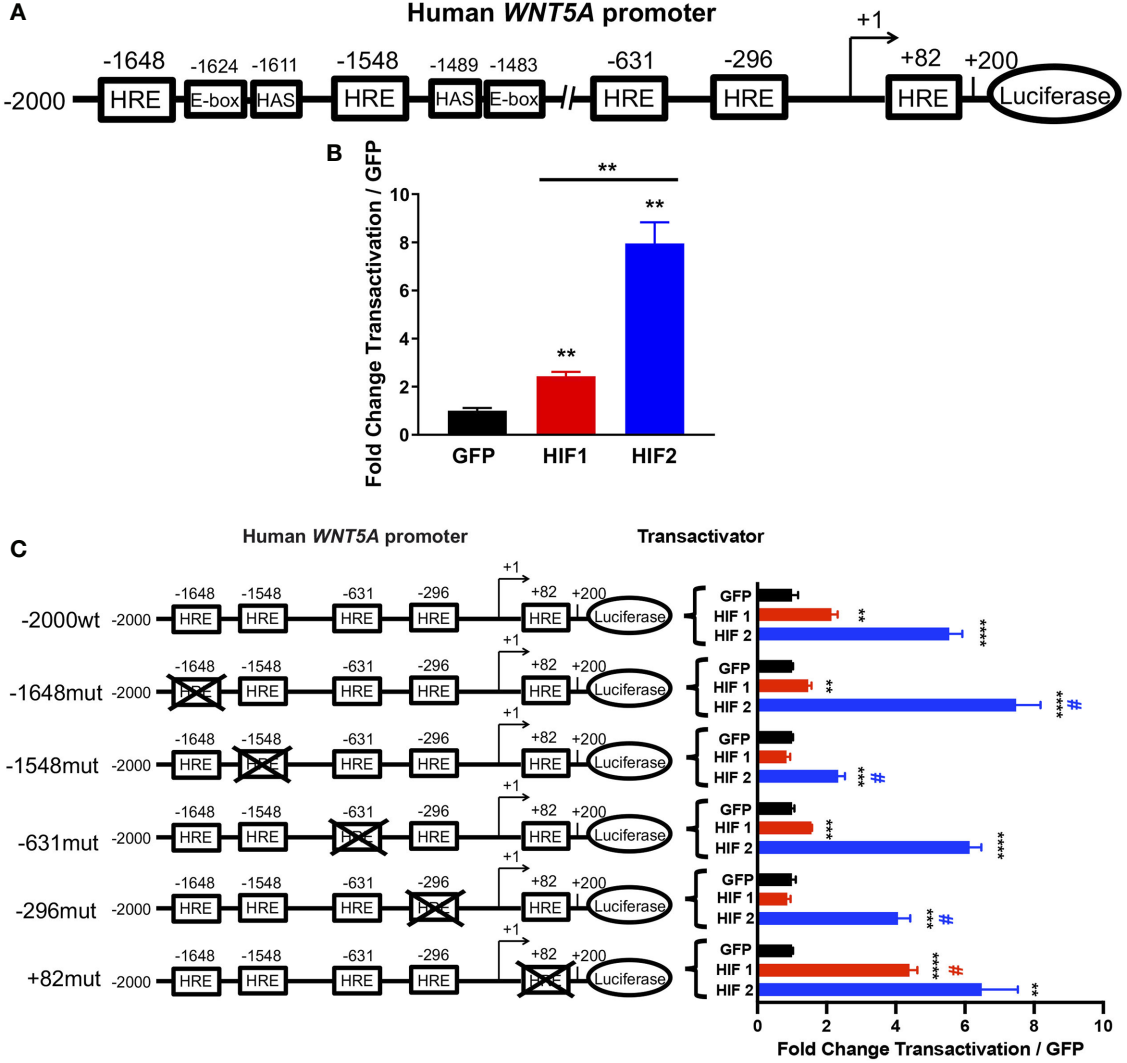
HIF2 directly activates the *WNT5A* promoter *via* HREs. **(A)** Human *WNT5A* promoter scheme showing distal (-1648, -1548, and -631) and proximal (-296 and +82) HREs identified by sequence analysis. **(B)** Dual luciferase reporter assay in AD-293 cells with wild-type human *WNT5A* promoter-luciferase construct and transactivation by HIF1, HIF2, or GFP control plasmids (n = 4 transfections/group). **(C)** Dual luciferase reporter assay in AD-293 cells with human *WNT5A* promoter-luciferase construct containing wild-type sequence (-2000wt) or mutations of distal (-1648mut, -1548mut, -631mut) and proximal (-296mut and +82mut) HREs with transactivation by HIF1, HIF2, or GFP control plasmids (n = 4 transfections/group). All error bars represent mean ± SEM. Asterisks indicate comparison of HIF1 or HIF2 to their respective GFP controls. **P < 0.01, ***P < 0.001, ****P < 0.0001, by Student's t test with False Discovery Rate two-stage step-up approach. # indicates comparison of construct transactivation in wild-type sequence to mutated sequences by HIF1 (red #) or by HIF2 (blue #); P < 0.05, by two-way ANOVA.

The HIFs are transcription factors that recognize and bind hypoxia response elements (HREs) in promoter or enhancer regions to induce gene transcription (13). Thus, we analyzed the *WNT5A* promoter sequence to determine whether it contained putative HRE motifs and identified multiple low- and high-stringency HRE consensus sequences (**Figure 4A** and **Supplemental Figure 2**). To determine whether induction by HIF2 occurs directly or indirectly, we designed luciferase reporter constructs of the human *WNT5A* promoter spanning from 2,000 nucleotides upstream of the transcriptional start site (34) to 200 nucleotides downstream, containing five HRE consensus sequences that are closely associated with HIF ancillary sequences (HAS) and E-box motifs (**Figure 4A** and **Supplemental Figure 2**). HAS and E-box motifs are cis-element that are required for an HRE to be functionally active. Both motifs play a role in the recruitment of transcriptional machinery that together with HIF2 induce promoter activation (37–39).We transfected this construct into human embryonic kidney-derived Adherent 293 (AD-293**)** cells along with an expression vector encoding GFP or constitutively active human HIF1 or HIF2 (11) and performed dual luciferase reporter assays (25). We found that HIF2 significantly increased *WNT5A* promoter transactivation by eight-fold over GFP controls (**Figure 4B**). HIF1 only modestly affected promoter activity (**Figure 4B**).

To understand if any specific HRE motif of the *WNT5A* promoter is required for its activation by HIF2, we performed mutational analyses. We engineered transversion point mutations on the guanine and cytosine nucleotides of three distal and two proximal putative HRE sites within the promoter, and transfected AD-293 cells with these constructs to perform promoter activation studies (**Figure 4C** and **Supplementary Figure 2**). We found that independently mutating the two most distal HREs in the *WNT5A* promoter had the most impact on HIF2-induced transactivation, and that these two HREs are differentially regulated by HIF2. The point mutation at the HRE in position -1548 significantly diminished the previously observed HIF2-induced promoter activation, suggesting that the -1548HRE is necessary for HIF2’s ability to bind and positively regulate the *WNT5A* promoter (**Figure 4C**). Importantly, this -1548HRE resides upstream of a HAS, which is also in close proximity to an E-box (**Figure 4A** and **Supplementary Figure 2**). Conversely, mutating the -1648HRE resulted in an increased ability of HIF2 to transactivate the *WNT5A* promoter, suggesting that it might be a repressive HRE. Although the HRE at -1648 resides nearby E-box and HAS motifs, HRE sites that are preceded by cytosine nucleotides on 5’ form E-box binding sites of other basic helix-loop-helix transcription factor families, and HIFs rarely recognize these (38). Moreover, while HIF2-induced gene upregulation is largely mediated by direct HIF2 binding to DNA motifs, HIF2-induced gene repression tends to occur indirectly through transcriptional co-repressors (37). Thus, we posit that direct binding of HIF2 to the -1548HRE promotes *WNT5A* transcription, whereas HIF2 interaction with the -1648HRE promotes the recruitment of repressive transcriptional machinery. The point mutations of the HREs at positions -631, -296, and +82 did not alter the activation of the WNT5A promoter by HIF2 (**Figure 4C**). Taken all together, our results indicate that HIF2 directly activates the *WNT5A* promoter, possibly by binding the -1548HRE.

### Wnt5a Increases ISC Survival and Cryptogenic Potential Following Radiation

**Figure 5:**
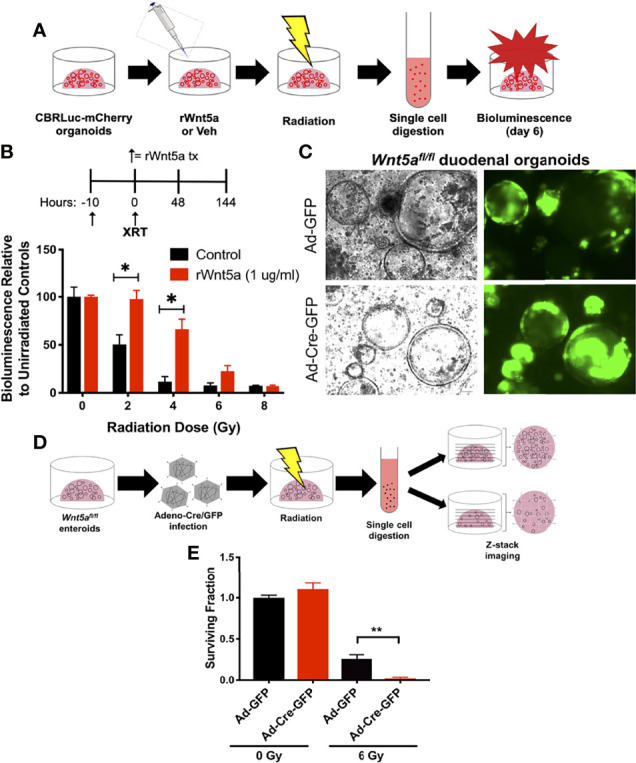
Wnt5a mediates HIF2-associated radioprotection of the small intestine. **(A)** Schematic representation illustrating how spheroid formation assays were performed using murine duodenal CBRLuc-mCherry reporter organoids to test whether rWnt5a radioprotects. **(B)** CBRLuc-mCherry organoids treated with vehicle (PBS) or 1 μg/ml rWnt5a for 10h were exposed to the indicated doses of ionizing radiation. Bioluminescence was measured 6 days post-radiation (n = 3 per group). **(C)** Wnt5a^fl/fl^ duodenal organoids were transduced with Adeno-Cre-GFP or control Adeno-GFP to generate Wnt5a knockouts. Representative bright field and GFP images are shown. **(D)** Schematic representation illustrating how spheroid formation assays were performed using Wnt5a^fl/fl^ duodenal organoids to test whether Wnt5a is necessary for crypt regeneration following radiation. **(E)** Wnt5a^fl/fl^ duodenal organoids infected with Adeno-Cre-GFP or Adeno-GFP were exposed to the indicated doses of ionizing radiation. Z-stack images were stitched and organoids larger than 150 μm in diameter were quantified 6 days post-radiation and mean surviving fraction is plotted (n = 3 per group). All error bars represent mean ± SEM. *P < 0.05, **P < 0.01, by Student’s t test.

Wnt5a is a non-canonical Wnt ligand that has been shown to promote the formation of new intestinal crypts in order to re-establish homeostasis after intestinal mucosal injury (26). To evaluate whether Wnt5a mediates HIF2-afforded GI radioprotection, we performed a spheroid formation assay, which is an *ex vivo* microcolony assay that allows us to evaluate potential radiation modulators in small intestinal organoid cultures (28). We treated CBRLuc-mCherry, a murine duodenal mCherry reporter organoid line (28), with recombinant Wnt5a (rWnt5a) 10 hours prior to irradiation, then exposed them to 0-8Gy of Xrays, and re-seeded single cells in Matrigel (**Figures 5A, B**). CBRLuc-mCherry organoids that were pre-treated with rWnt5a produced significantly higher relative bioluminescence levels six days after irradiation, compared to vehicle-treated organoids, indicating that rWnt5a increased the number of regenerating crypts (**Figure 5B**). Pre-treatment with rWnt5a significantly increased the cryptogenic capacity of CBRLuc-mCherry organoids exposed to 2 Gy and 4 Gy by 2-fold and 6-fold, respectively, and also increased the cryptogenic capacity of organoids exposed to 6 Gy by 2.8-fold, but this was not statistically significant (**Figure 5B**). Interestingly, CBRLuc-mCherry organoids that continued receiving rWnt5a treatment after irradiation did not display improved cryptogenic capacity (**Supplementary Figure 3A**). Moreover, initiating rWnt5a treatments only after irradiation also did not improve the cryptogenic capacity of CBRLuc-mCherry organoids (**Supplementary Figure 3B**). These results suggest that Wnt5a could be radioprotective to small intestinal crypts but does not mitigate radiation damage after it has occurred. A possible explanation for these results is that Wnt5a suppresses intestinal organoid proliferation by inducing TGF-β signaling (26), which would protect cycling ISCs from radiation, but would dampen their capacity to regenerate crypts if Wnt5a treatment was continued after the radiation injury was incited.

**Figure 6:**
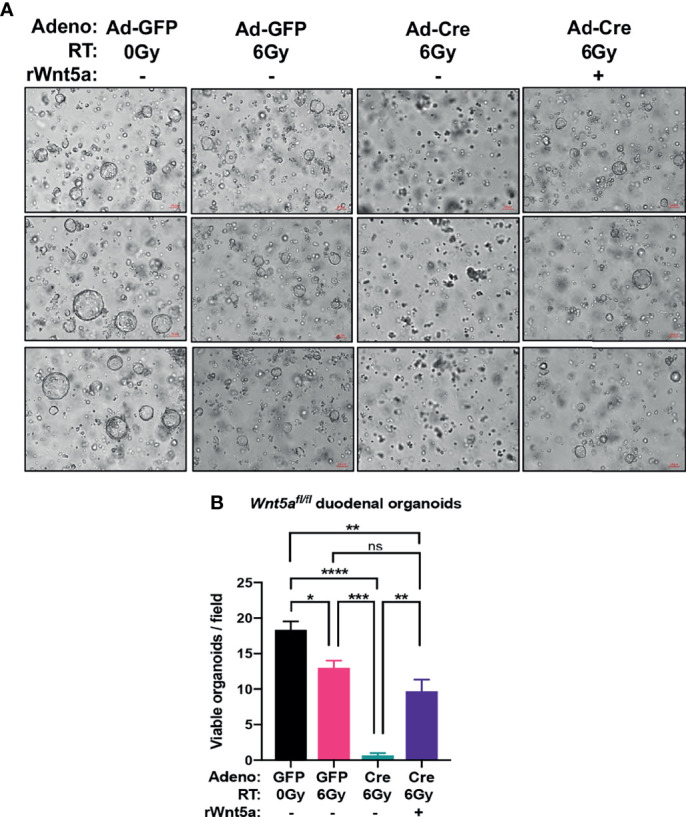
rWnt5a treatment rescues crypt regeneration in irradiated *Wnt5a^CKO^* duodenal organoids. *Wnt5a^fl/fl^* organoids were infected with Adeno-Cre-GFP or control Adeno-GFP, treated with vehicle or rWnt5a (600 ng/ml), and then treated with the indicated doses of ionizing radiation. **(A)** Representative bright field images with **(B)** quantification of viable organoids are shown 6 days after irradiation (n = 3 per group). All error bars represent mean ± SEM. *P < 0.05, **P < 0.01, ***P < 0.001, ****P < 0.0001, and ns = not significant, by Tukey’s multiple comparisons test" instead of "*P < 0.05, **P < 0.01, ***P < 0.001, ****P < 0.0001, and ns, not significant, by Tukey's multiple comparisons test.

To understand whether Wnt5a is necessary for intestinal crypt regeneration following radiation injury, we generated conditional *Wnt5a* knockout (*Wnt5a^CKO^*) duodenal organoids from *Wnt5a^fl/fl^* mice and transduced them with Adeno-Cre-GFP or control Adeno-GFP vectors. In agreeance with published work demonstrating that Wnt5a is dispensable for homeostasis in the gut postnatally (40), deletion of Wnt5a did not affect organoid growth or morphology (**Figure 5C**). We then performed a modified spheroid formation assay using the *Wnt5a^CKO^* duodenal organoids and z-stack imaging, rather than bioluminescence, to quantify regenerating organoids (**Figure 5D**). Loss of Wnt5a did not affect the cryptogenic capacity of unirradiated duodenal organoids (**Figure 5E** and **Supplementary Figure 4**), again confirming that *Wnt5a* is dispensable for crypt homeostasis (**Figure 5C**) (40). On the other hand, deletion of *Wnt5a* significantly reduced the fraction of surviving duodenal organoids after 6 Gy of radiation (**Figure 5E** and **Supplementary Figure 4**), indicating that Wnt5a is necessary for crypt regeneration following radiation. Furthermore, treatment with recombinant Wnt5a (rWnt5a) rescued Wnt5a-depleted duodenal organoids from radiation-induced ISC death (**Figure 6**). Taken together, these results suggest that Wnt5a increases ISC survival and is both necessary and sufficient for ISC radioprotection.

## Discussion

Radiation therapy for abdominal and pelvic tumors is challenging because the small intestine is exquisitely radiosensitive, which limits the dose that can be delivered to tumors without major GI toxicity (2–6). Unfortunately, there are no FDA-approved therapies to prevent GI radiotoxicities. Multiple groups, including our own, have shown that HIF2 stabilization by pharmacological EGLN inhibition protects the small intestine against radiation (22, 23, 41, 42). However, the mechanism by which HIF2 radioprotects ISC and prevents GI radiotoxicity remains unclear. The current study provides mechanistic insight into how HIF2 reduces the radiosensitivity of the intestinal crypt. Moreover, to the best of our knowledge, this is the first study that evidences a convergence between non-canonical Wnt signaling and hypoxia signaling.

Here we used ISC-enriched 3D duodenal organoid cultures to mimic the small intestinal crypt and studied the mechanism of HIF2 radioprotecion by both transient and stable HIF2 overexpression. Unsurprisingly, the HIF2-induced intestinal transcriptome included many genes that are essential for normal GI homeostasis and barrier function, and interestingly, some of these genes have been implicated in cellular response to radiation. We took a candidate approach and focused our attention on Wnt5a, which has been shown to play roles in the development of the intestinal tract, the proliferation of ISCs, and their capacity to regenerate upon GI injury (26, 40, 43). In zebrafish embryos, Wnt5a has also been shown to regulate gastrulation and to ameliorate radiation-induced toxicity (43). Here we provide evidence that Wnt5a is a direct HIF2 target. We identified five major HRE sites within the human *WNT5A* promoter and our experiments in human cells confirmed that HIF2 directly activates the *WNT5A* promoter through a functional HRE motif located 1548 nucleotides upstream of the transcriptional start site. This -1548HRE site resides near HAS and E-box sequences, which help recruit transcriptional co-activators (38). Nevertheless, our promoter studies were focused on 2,2000 nucleotides surrounding the *WNT5A* transcriptional start site, thus, there could be additional distal HRE sites that are regulated by HIF2.

Our study is the first to show that Wnt5a could be a potential target to prevent GI radiotoxicity. We showed that Wnt5a is both necessary and sufficient for ISC survival and crypt regeneration after exposure to radiation. Deletion of *Wnt5a* completely impaired the ability of ISCs to form crypt spheres after being irradiated, and addition of rWnt5a rescued ISCs from radiation-induced cell death. Importantly, our phenotype was only reproducible when rWnt5a was administered before radiation treatments. Wnt5a signaling has been shown to inhibit both intestinal and hematopoietic stem cell proliferation (26, 44). Because radiation is more toxic to rapidly proliferating cells, suppression of cell proliferation prior to radiation would allow ISCs to sustain the effects of radiation. Wnt5a binds to the Frizzled (Fzd) family of cell surface receptors, including Fzd-1/2/4/5/7/8 (45), and its canonical co-receptors Lrp5/6 or its non-canonical co-receptors Ror1/2 and Ryk, and activates either the canonical Wnt/Beta-catenin pathway, the non-canonical planar cell polarity pathway, or the non-canonical Wnt/Ca^2+^ pathways (34). In intestinal organoids, non-canonical Wnt5a signaling through Ror2 induces TGF-β signaling and Smad3 phosphorylation with subsequent nuclear translocation, leading to increased expression of multiple cyclin-dependent kinase inhibitors, and ultimately arrest of cell proliferation (26). Thus, activation of these downstream signaling pathways could be a possible explanation for the relative success of Wnt5a as a radioprotector while failing to mitigate radiation injury post-exposure. It is important to note that our studies were limited to *ex vivo* duodenal organoid models and that our observations should be validated using *in vivo* GI radiation models.

Wnt5a has been implicated in tumor progression, raising concerns about its potential use as a GI radioprotective agent in cancer patients (46, 47). While this concern is warranted, HIF2 would only be activated for a short period only during radiation to reduce normal tissue toxicity, which may reduce the potential oncogenic effects of this molecule. Additional pre-clinical studies using cancer models to assess this relative risk are warranted before Wnt5a can be considered for clinical translation. Furthermore, we note that our radiation model employs conventional single fractions, whereas many GI radiation oncology regimens employ fractionated radiation. Accordingly, the extent of Wnt5a-afforded ISC radioprotection would need to be evaluated in the setting of fractionated regimens.

We note that HIF2 may have additional molecular mechanisms by which it promotes ISC survival and intestinal radioprotection. Prior studies have shown that HIF2 both protects and mitigates GI radiation injury (22), yet our results here suggest that Wnt5a does not mitigate intestinal crypt radiation injury. Thus, future studies should assess which HIF2 targets are potential GI radiation mitigators. For example, the *Neuroepithelial cell transforming 1* (*Net1*) gene, which was significantly upregulated in our HIF2 dataset, has been shown to play a role in DNA damage repair after ionizing radiation (48, 49). Similarly, *Ets1* and *Klf4* are transcription factors that are essential for stem cell self-renewal and can regulate DNA damage repair (50, 51), and both were significantly upregulated by HIF2.

Moreover, our study was limited to the ISC compartment of the small intestine. However, other cellular compartments, such as the intestinal stromal niche, gut macrophages and the endothelial compartment have established roles in the intestinal response to injury (52, 53). Further investigation into these cellular compartments is required to fully dissect the role of HIF2 in the radiation responses of the intestinal tract.

## Data Availability Statement

The data presented in this study are deposited in the GEO repository, accession number GSE186927.

## Ethics Statement

The animal study was reviewed and approved by the Institutional Animal Care and Use Committee of The University of Texas MD Anderson Cancer Center.

## Author Contributions

Conceptualization, CG and CT. Data Curation, CG and SG. Formal Analysis, CG, AA, NK, SG, MD, and FL. Funding Acquisition, CG and CT. Investigation, CG, AA, and SG. Methodology, CG, SG, MD, HP-W, and CT. Project Administration, CG, SG, and CT. Resources, HP-W, AM, and CT. Software, FL and NN. Supervision, CT. Validation, CG, AA, NK, NN, and CT. Visualization, CG, AA, SG, NK, MD, and FL. Writing – Original Draft, CG, AA, NK, IJ, and CT. Writing – Review and Editing, CG, IJ, and CT. All authors contributed to the article and approved the submitted version.

## Funding

CT was supported by funding from the National Institutes of Health (NIH) under award number R01CA227517-01A1 and GI SPORE grant (P50CA221707), by the Cancer Prevention & Research Institute of Texas (CPRIT) under grant RR140012, by the V Foundation (V2015-22), by the Sidney Kimmel Foundation, by a Sabin Family Foundation Fellowship, by the Reaumond Family Foundation, by the Mark Foundation, by the Childress Family Foundation, by the McNair Family Foundation, and by generous philanthropic contributions to The University of Texas MD Anderson Moon Shots Program. CG was supported by the National Institute of Diabetes, Digestive and Kidney Diseases (NIDDK) of the NIH under award number F31DK121384. MD was supported by the National Cancer Institute (NCI) of the NIH under award number F31CA210631. CG, MD, AA, and IS were also supported by the NIH/NCI under award number U54CA096300/297. This work was also supported in part by NIH/NIDDK grant DK056338, which supports the Texas Medical Center Digestive Diseases Center, and by NIH/NCI Cancer Center Support Grants (CCSG) P30CA016672, which supports MDACC’s Sequencing and Microarray Facility.

## Conflict of Interest

CT is on the clinical advisory board of Accuray, as well as has a patent for oral amifostine as a radioprotectant of the upper GI tract issued, licensed, and with royalties paid from Xerient Pharmaceuticals and PHD inhibitors as a radioprotectant of the GI tract pending, and was the lead principal investigator of a multicenter trial testing the effects of high-dose SBRT with the radiomodulator, GC4419. CT is also a paid consultant for Phebra Pty, Ltd.

The remaining authors declare that the research was conducted in the absence of any commercial or financial relationships that could be construed as a potential conflict of interest.

## Publisher’s Note

All claims expressed in this article are solely those of the authors and do not necessarily represent those of their affiliated organizations, or those of the publisher, the editors and the reviewers. Any product that may be evaluated in this article, or claim that may be made by its manufacturer, is not guaranteed or endorsed by the publisher.

## Supplementary Material

Supplementary Table 1 Primer Assays used for qRT-PCR.

Supplementary Table 2 Differentially Expressed Genes in HIF2-overexpessing duodenal organoids versus wild-type duodenal organoids.

Supplementary Table 3 Differentially Expressed Genes in HIF1-overexpessing duodenal organoids versus wild-type duodenal organoids.

Supplemental Figure 1 Differentially expressed gene profile in HIF1-overexpressing duodenal organoids. **(A)** Heatmap shows all the differentially expressed genes in duodenal organoids infected with Adeno-hHIF1 or control Adeno-GFP evaluated via RNA sequencing (n = 3 biological replicates/group). **(B)** Volcano plot of the differentially expressed genes.

Supplementary Figure 2 Sequences of the Human WNT5A promoter WT and HRE mutant constructs used for this study.

Supplementary Figure 3 Wnt5a does not a mitigate radiation-induced intestinal crypt cell death. **(A)** (CBRLuc-mCherry duodenal reporter organoids treated with vehicle (PBS) or 1 μg/ml rWnt5a for 10h were exposed to the indicated doses of ionizing radiation, and then were treated with rWnt5a again immediately after the spheroid formation assay was seeded, and 48h later. Bioluminescence was measured 6 days post-radiation (n = 3 per group). Continuous rWnt5a treatment does not improve clonogenic capacity of CBRLuc-mCherry organoids exposed to radiation. **(B)** CBRLuc-mCherry duodenal reporter organoids were exposed to the indicated doses of ionizing radiation, and then were treated with vehicle (PBS) or 1 μg/ml rWnt5a for 5h and 48h after irradiation. Bioluminescence was measured 6 days post-radiation (n = 3 per group). Post-radiation rWnt5a treatment does not improve clonogenic capacity of CBRLuc-mCherry organoids. All error bars represent mean ± SEM. *P < 0.05, by Student’s t test.

Supplementary Figure 4 Wnt5a is necessary for crypt regeneration in response to radiation. Wnt5afl/fl duodenal organoids infected with Adeno-Cre-GFP or Adeno-GFP and were then exposed to the indicated doses of ionizing radiation. Z-stack images were stitched and organoids larger than 150 μm in diameter were quantified 6 days post-radiation (see Figure 5E for mean surviving fraction values; n = 3 per group). Representative bright field Z-stack images are shown.
