# Supplementary figures and images for: HIF2 Regulates Intestinal Wnt5a Expression
Source: Front Oncol. 2021 Nov 25;11:769385. doi: 10.3389/fonc.2021.769385 (PMC8656274; doi:10.3389/fonc.2021.769385)

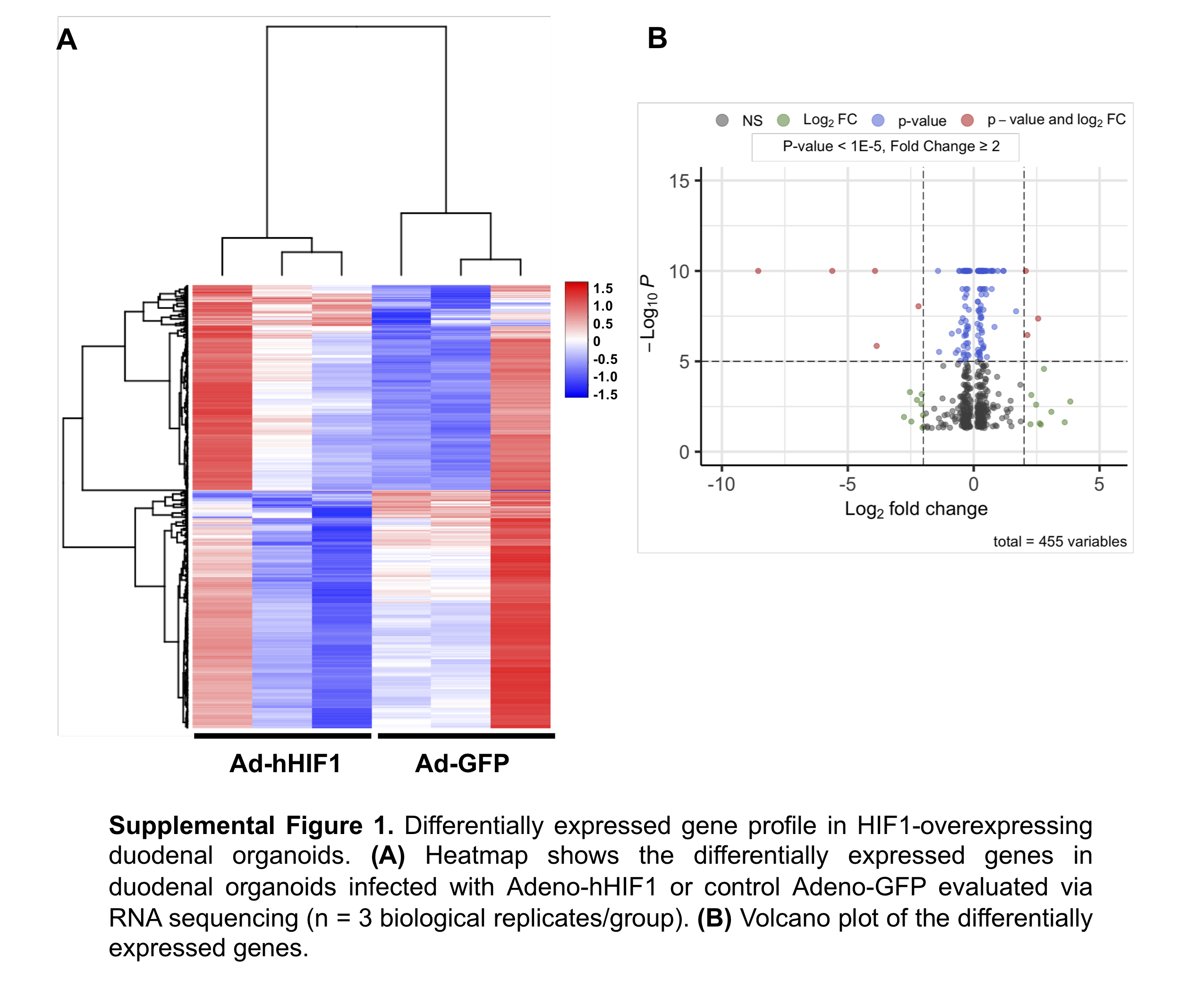

Supplement: Supplemental Figure 1 — Differentially expressed gene profile in HIF1-overexpressing duodenal organoids. (A) Heatmap shows all the differentially expressed genes in duodenal organoids infected with Adeno-hHIF1 or control Adeno-GFP evaluated via RNA sequencing (n = 3 biological replicates/group). (B) Volcano plot of the differentially expressed genes. [file Image_1.tiff]

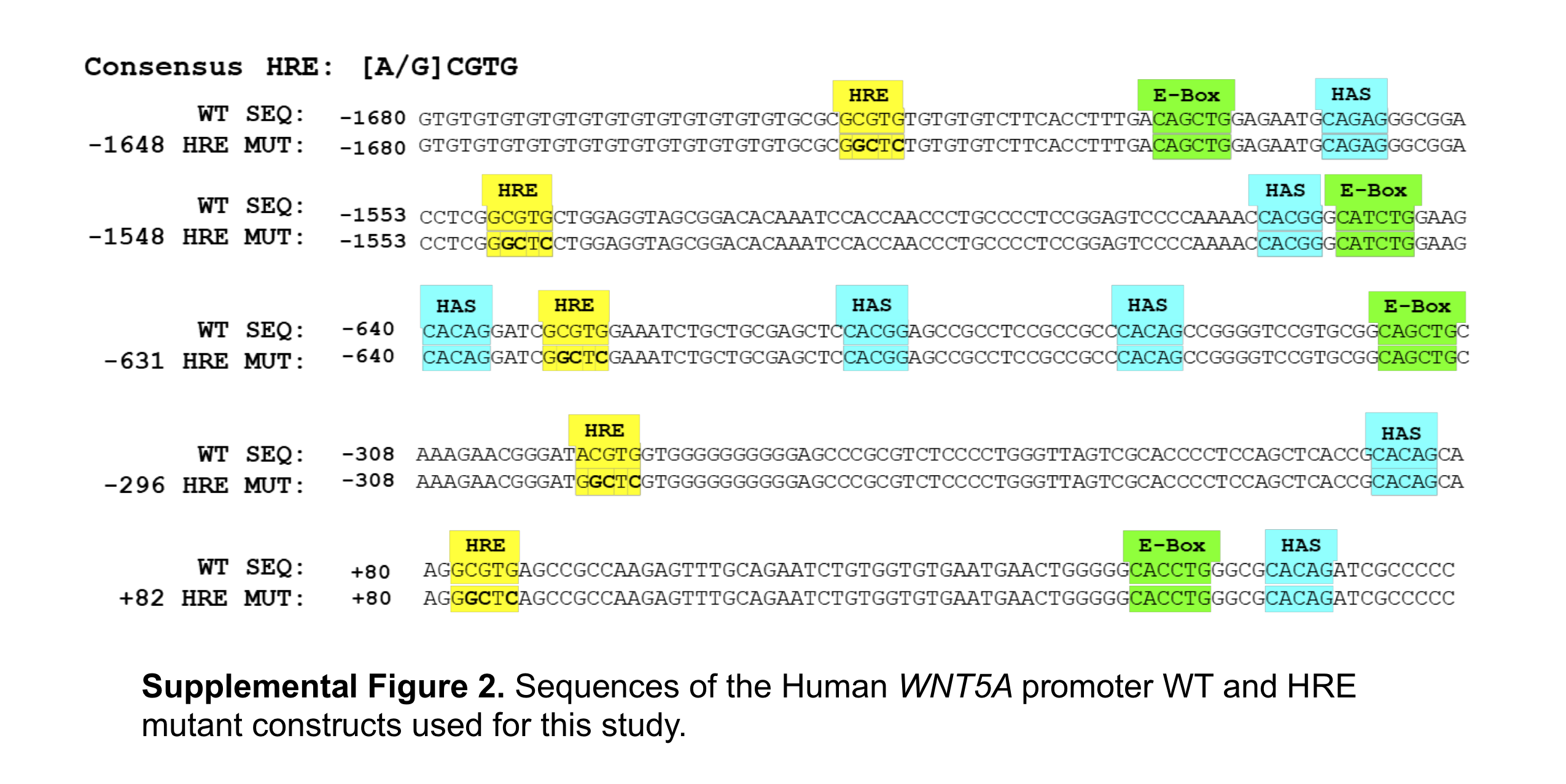

Supplement: Supplementary Figure 2 — Sequences of the Human WNT5A promoter WT and HRE mutant constructs used for this study. [file Image_2.tiff]

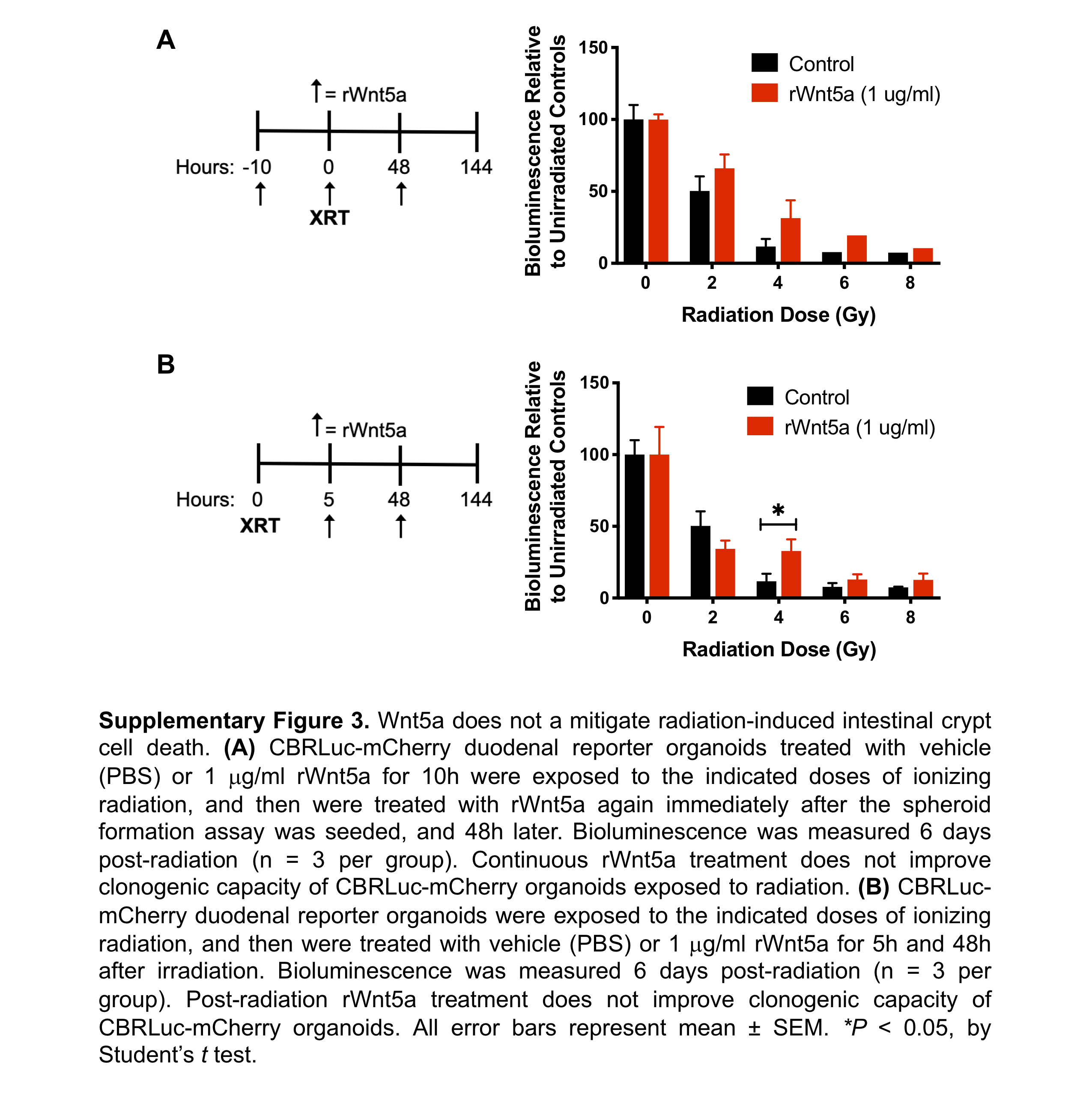

Supplement: Supplementary Figure 3 — Wnt5a does not a mitigate radiation-induced intestinal crypt cell death. (A) (CBRLuc-mCherry duodenal reporter organoids treated with vehicle (PBS) or 1 μg/ml rWnt5a for 10h were exposed to the indicated doses of ionizing radiation, and then were treated with rWnt5a again immediately after the spheroid formation assay was seeded, and 48h later. Bioluminescence was measured 6 days post-radiation (n = 3 per group). Continuous rWnt5a treatment does not improve clonogenic capacity of CBRLuc-mCherry organoids exposed to radiation. (B) CBRLuc-mCherry duodenal reporter organoids were exposed to the indicated doses of ionizing radiation, and then were treated with vehicle (PBS) or 1 μg/ml rWnt5a for 5h and 48h after irradiation. Bioluminescence was measured 6 days post-radiation (n = 3 per group). Post-radiation rWnt5a treatment does not improve clonogenic capacity of CBRLuc-mCherry organoids. All error bars represent mean ± SEM. *P < 0.05, by Student’s t test. [file Image_3.tiff]

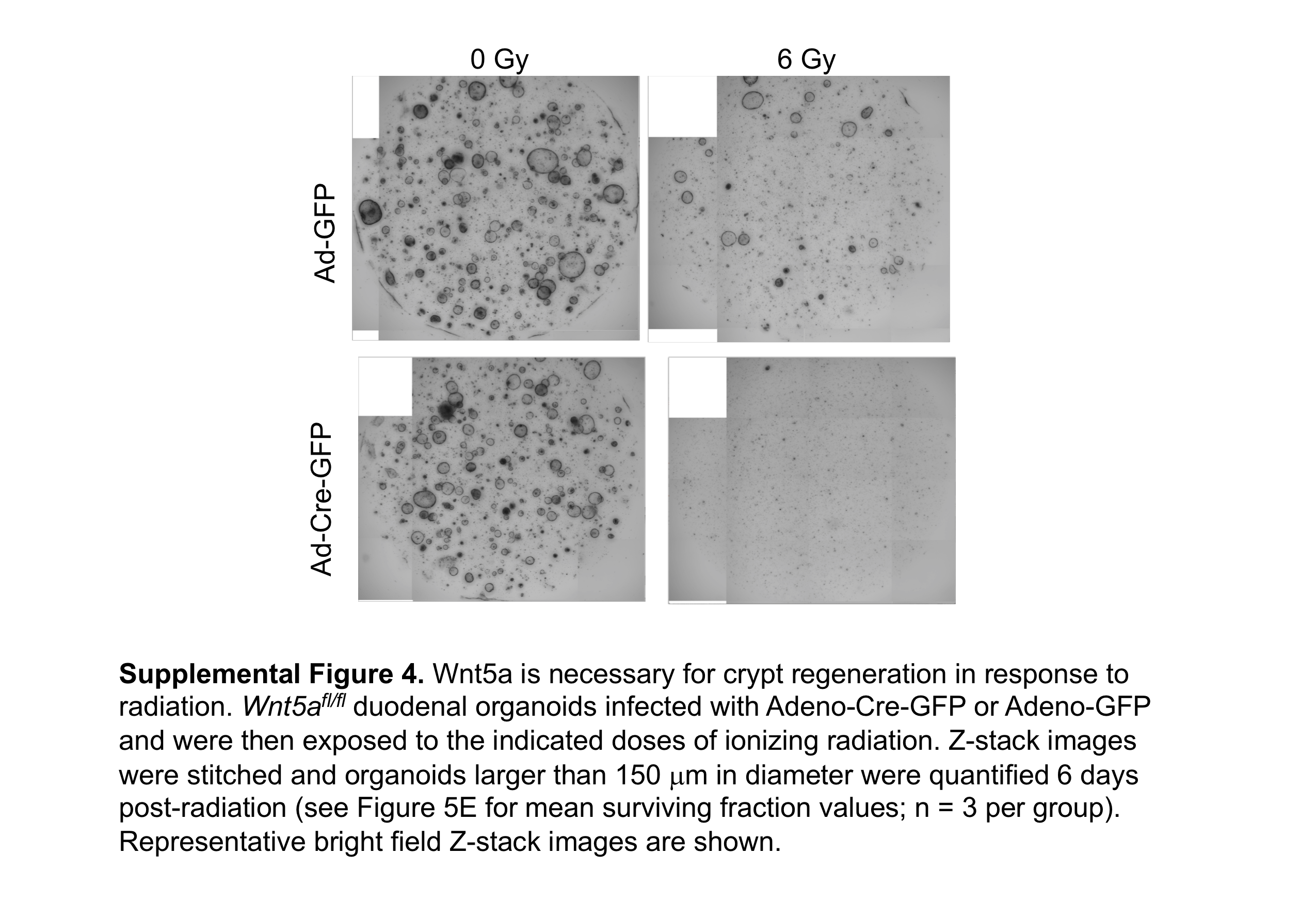

Supplement: Supplementary Figure 4 — Wnt5a is necessary for crypt regeneration in response to radiation. Wnt5afl/fl duodenal organoids infected with Adeno-Cre-GFP or Adeno-GFP and were then exposed to the indicated doses of ionizing radiation. Z-stack images were stitched and organoids larger than 150 μm in diameter were quantified 6 days post-radiation (see Figure 5E for mean surviving fraction values; n = 3 per group). Representative bright field Z-stack images are shown. [file Image_4.tiff]
